# Thrombus aspiration in hyperglycemic ST-elevation myocardial infarction (STEMI) patients: clinical outcomes at 1-year follow-up

**DOI:** 10.1186/s12933-018-0795-8

**Published:** 2018-11-29

**Authors:** Celestino Sardu, Michelangela Barbieri, Maria Luisa Balestrieri, Mario Siniscalchi, Pasquale Paolisso, Paolo Calabrò, Fabio Minicucci, Giuseppe Signoriello, Michele Portoghese, Pasquale Mone, Davide D’Andrea, Felice Gragnano, Alessandro Bellis, Ciro Mauro, Giuseppe Paolisso, Maria Rosaria Rizzo, Raffaele Marfella

**Affiliations:** 1 Department of Medical, Surgical, Neurological, Aging and Metabolic Sciences, University of Campania “Luigi Vanvitelli”, Piazza Miraglia, 2, 80138 Naples, Italy; 2 Department of Biochemistry, Biophysics and General Pathology, University of Campania “Luigi Vanvitelli”, Naples, Italy; 3 Department of Cardiology, Hospital Cardarelli, Naples, Italy; 4 Department of Cardio-Thoracic and Respiratory Sciences, University of Campania “Luigi Vanvitelli”, Caserta, Italy; 5 Department of Mental Health and Public Medicine, Section of Statistic, University of Campania “Luigi Vanvitelli”, Naples, Italy; 6 Department of Cardiac Surgery, Hospital “SS Annunziata”, Sassari, Italy

**Keywords:** Hyperglycemia, STEMI, Primary percutaneous coronary intervention, Thrombus aspiration

## Abstract

**Objectives:**

We evaluate whether the thrombus aspiration (TA) before primary percutaneous coronary intervention (PPCI) may improve STEMI outcomes in hyperglycemic patients.

**Background:**

The management of hyperglycemic patients during STEMI is unclear.

**Methods:**

We undertook an observational cohort study of 3166 first STEMI. Patients were grouped on the basis of whether they received TA or not. Moreover, among these patients we selected a subgroup of STEMI patients with hyperglycemia during the event (glycaemia > 140 mg/dl). The endpoint at 1 year included all-cause mortality, cardiac mortality and re-hospitalization for coronary disease, heart failure and stroke.

**Results:**

One-thousand STEMI patients undergoing PPCI to plus TA (TA-group) and 1504 STEMI patients treated with PPCI alone (no-TA group) completed the study. In overall study-population, Kaplan–Meier-analysis demonstrated no significant difference in mortality rates between patients with and without TA (P = 0.065). After multivariate Cox-analysis (HR: 0.94, 95% CI 0.641–1.383) and the addition of propensity matching (HR: 0.86 95% CI 0.412–1.798) TA was still not associated with decreased mortality. By contrast, in hyperglycemic subgroup STEMI patients (TA-group, n = 331; no-TA group, n = 566), Kaplan–Meier-analysis demonstrated a significantly lower mortality (P = 0.019) in TA-group than the no-TA group. After multivariate Cox-analysis (HR: 0.64, 95% CI 0.379–0.963) and the addition of propensity matching (HR: 0.54, 95% CI 0.294–0.984) TA was still associated with decreased mortality.

**Conclusions:**

TA was not associated with lower mortality in PPCI for STEMI when used in our large all-comer cohort. Conversely, TA during PPCI for STEMI reduces clinical outcomes in hyperglycemic patients.

*Trial registration* NCT02817542. 25th, June 2016

**Electronic supplementary material:**

The online version of this article (10.1186/s12933-018-0795-8) contains supplementary material, which is available to authorized users.

## Introduction

Admission hyperglycemia (AH) is a common finding among patients diagnosed with acute myocardial infarction (AMI), and is an independent predictor of short- and long-term mortality in patients with and without diabetes [1, 2]. Despite primary percutaneous coronary intervention (PPCI) is the mainstay of treatment in patients with ST-segment elevation myocardial infarction (STEMI) [3] the incidence of restenosis [4], heart failure, re-infarction and death in hyperglycemic STEMI patients remains significant [5]. Re-stenosis and no-reflow are common adverse events leading to worse clinical outcomes in hyperglycemic patients with STEMI undergoing primary PPCI, with athero-thrombotic embolization, increase of oxidative stress and inflammation as the main responsible mechanisms [6–8]. In an attempt to abrogate the increased mortality from AH, coronary care units have implemented insulin protocols to normalize glucose levels during AMI [2]. Treatment with insulin itself has shown mixed results [9]. Data from small pilot studies, moderate-sized clinical trials, and a meta-analysis suggested benefits of treatment with insulin [6–8, 10–12]. However, subsequent larger randomized control trials have failed to confirm improved survival [12–15]. Therefore, the management of hyperglycemic patients with STEMI in the context of contemporary revascularization remains unclear [16]. In this context, thrombus aspiration (TA) during primary PPCI has been thought to be an effective method for reducing distal embolization and improving microvascular perfusion [17]. However, randomized controlled trials [18] and large [19] trial registry studies show conflicting results of TA as a routine treatment and in operator selected patients in STEMI during PPCI. Moreover, the largest trials in both categories now firmly point in the direction of no clinical benefit of routinely using adjunctive TA in the treatment of STEMI patients undergoing PPCI, in general population [20]. However, the impact of TA on a sub-group population such as the hyperglycemic patients with STEMI was not addressed in any of these trials. A large body of evidence suggest that hyperglycemia causes over production of reactive oxygen species and inflammation from atherosclerotic plaque as well as from thrombus plaque, favouring athero-thrombotic embolization and poor myocardial infarction outcomes [21]. Thus, TA before PPCI can theoretically protect the peri-infarct myocardium from the excess of inflammation and oxidative stress resulting as an effect of hyperglycemia on the thrombus. Therefore, we conducted a study to evaluate the effects of TA on hard clinical end points in hyperglycemic patients with STEMI and compared those with the data obtained from a general population without differentiation between hyperglycemic and normoglycemic patients, as evaluated in the previous studies. Moreover, we compared pro-inflammatory/oxidative stress status of coronary thrombi in STEMI patients with and without hyperglycemia undergoing PPCI and adjunctive TA.

## Materials and methods

### Patients and study design

This was an observational prospective cohort study to investigate the relationship between TA use and outcome after PPCI for STEMI. We examined an observational cohort of consecutive patients with first STEMI treated with PPCI between February 2010, and July 2015 at the Department of Cardiology of the Cardarelli Hospital in Naples Italy, at Department of Cardiology of the University of Campania “Luigi Vanvitelli” Italy, and at Department of Cardiac Surgery of the SS. Annunziata Hospital, Sassari, Italy. Our observational study was designed to monitor and describe real life management/treatment of STEMI. All patients with onset of symptoms of < 12 h and at least 1-mm ST-segment elevation in two or more contiguous limb leads or at least 2 mm in two or more contiguous precordial leads or left bundle branch block were considered for PPCI. Coronary angiography was performed via the radial or femoral artery. The culprit lesion was identified and crossed with an angioplasty guidewire. Manual TA was performed at the discretion of the operator, as were technical aspects such as type and number of stents, use of any other devices, and with consideration of angiographic selection criteria (such as the presence of a visible thrombus on angiography), followed by conventional PCI to the culprit vessel. Inclusion criteria included: correspondence between ECG findings and suspected culprit artery; a minimum visual estimate of 50% stenosis in the culprit artery, and feasibility of performing TA, as judged by the treating physician; age of 18 years or greater; presentation to the cardiac catheterization laboratory for PPCI in the setting of first STEMI. Patients with left ventricular ejection fraction less than 25%, with previous myocardial infarction or previous PPCI or/and coronary by-pass grafting, or had received fibrinolytic therapy were excluded from the study. Patients were divided into two groups, on the basis of use of a TA during the PPCI (TA group), vs. no such use (no-TA group). Moreover, TA effects were analyzed on a sub-group of the same population consisting of hyperglycemic STEMI patients, presenting within 2 h after admission plasma glucose level of > 140 mg/dl as suggested by the position statement of the American Heart Association [2]. The investigation conforms with the principles outlined in the Declaration of Helsinki for use of human tissue or subjects. The Institutional Review Board of University of Campania “Luigi Vanvitelli” Italy approved the protocol.

### Procedures

Routine analyses were obtained on admission before coronary angiography and before full medical therapy was started. Treatment was classified as ‘‘thrombus aspiration’’ when TA was attempted as indicated by the operator, irrespective of procedure success. Thrombus grade was classified in accordance with Sianos et al. [22]: Grade 0 (G0), no angiographic characteristics of thrombus present; Grade 1 (G1), possible thrombus present, with the following angiography characteristics: reduced contrast density, haziness, irregular lesion contour, or a smooth convex meniscus at the site of total occlusion suggestive, but not diagnostic, of thrombus; Grade 2 (G2), definite thrombus with largest dimension ≤ 1/2 the vessel diameter; Grade 3 (G3), definite thrombus, with largest linear dimension > 1/2 but < twice vessel diameter; Grade 4 (G4), definite thrombus, with the largest dimension ≥ 2 vessel diameters; Grade 5 (G5), total occlusion, unable to assess thrombus burden due to total vessel occlusion. Detailed recommendations were provided about TA procedure. The procedure was done by investigators who were interventional cardiologists. All patients were treated with adenosine (given 120 mcg as a fast bolus followed by 2 mg in 2 min) and with bolus infusion of abciximab (0.25 mg/kg IV bolus). Patients undergoing TA received the same treatment after thrombus aspiration. Aspiration was to be started before crossing the lesion. A minimum of two syringes (40 ml) of aspirate were recommended. Investigators were instructed to ensure that the guide catheter was engaged with the coronary ostia when removing the thrombectomy catheter. Finally, the guide catheter was aspirated after thrombectomy to avoid embolization of air or thrombus from the guide catheter.

### Thrombus analysis

Thrombi obtained from 50 hyperglycemic and 50 normo-glycemic propensity score (PS) matched STEMI patients were analyzed for pro-inflammatory and oxidative stress status. All analyses were performed by 3 independent pathologists, blinded to the patient’s characteristics. After TA, the specimens were cut perpendicular to the long axis into two halves. The first half was frozen in liquid nitrogen for the following ELISA analysis. A portion of the other half of the specimen was immediately immersion fixed in 10% buffered formalin. Sections were serially cut at 4-μm thickness. Thrombi specimens were analyzed by light microscopy.

#### Immunohistochemistry

The sample was placed in 10% formalin immediately after retrieval and fixed for 24 h. The number of fragments recovered and the dimensions of each were recorded. The material was then entirely cut in 3-micra serial sections. In each obtained specimen, the percentage of leukocytes and fibrin was registered. Serial sections were incubated with the following specific antibodies: anti-CD68 (cluster of differentiation 68 glycoprotein) (Dako, Milan, Italy), markers of macrophages; anti-matrix metallopeptidase-9 (MMP-9) (Santa Cruz Biotechnology), tumor necrosis factor-α (TNF-α) (R&D Systems, Milan, Italy), and nitrotyrosine (Dako, Santa Clara, CA 95051, United States). Immunohistochemistry analysis was performed with a personal computer-based quantitative 24-bit color image analysis system (IM500, Leica Microsystem AG) to evaluate areas with higher vs. lower expression of the analyzed marker. However, these areas expressing in percentage these markers have been called as rich areas %.

#### Biochemical assays

Thrombi were lysed and centrifuged for 10 min at 10,000×*g* at 4 °C. After centrifugation, 20 mg of each sample was loaded onto a nitrocellulose membrane. Each determination was repeated at least three times. MMP-9, TNF-α, and nitrotyrosine levels were quantified in plaques using specific ELISA kits (from Santa Cruz Biotechnology, R&D Systems, and Abcam).

#### Blood glucose control in emergency wards

After coronary angiography procedures, all hyperglycemic patients with blood glucose ≥ 180 mg/dl were treated with intensive glucose control to keep blood glucose levels between 140 and 180 mg/dl, as previously described [4]. Continuous insulin infusion of 50 IU Actrapid HM (Novo-Nordisk) in 50 ml NaCl (0.9% using a Perfusor-FM-pump) was started only when blood glucose levels exceeded 180 mg/dl and adjusted to keep blood glucose between 140 and 180 mg/dl. When blood glucose fell < 140 mg/dl, insulin infusion were tapered and eventually stopped. After the start of insulin infusion protocol a glycemic control was provided every hour in order to obtain three consecutive values that were within the goal range. The infusion lasted until stable glycemic goal (140–180 mg/dl) for at least 24 h was reached. After that glycemic goal was maintained for 24 h, the infusion was stopped and subcutaneous insulin was initiated. Insulin was given as short-acting insulin before meals and long-acting insulin in the evening throughout the period of hospital stay. With regard to the full medical therapy, the protocol stated that the use of concomitant treatment should be as uniform as possible and accorded to evidence-based international guidelines for STEMI [23].

### Follow-up

After discharge from the hospital, all patients were managed and followed quarterly for 12 months after event, as outpatients, to perform clinical evaluation, routine analyses and cardiovascular evaluation (ECG, exercise ECG, echocardiography), as well as, with the goal to maintain HbA1c level at < 7% in diabetic patients.

### Outcomes

The primary outcome of the “thrombus aspiration in hyperglycemic ST-elevation myocardial infarction (STEMI) patients: clinical outcomes at 1-year follow-up” (TAHITI) study was all-cause mortality and cardiovascular death. Other outcomes including re-hospitalization for acute coronary syndrome class IV heart failure and stroke within 360 days.

### Statistical analyses

Clinical characteristics of TA vs. PPCI-only treated patients were compared using the Pearson Chi square test for categorical variables and Student t test for continuous variables, in overall study population and the sub-group of hyperglycemic patients. Normality of distribution was assessed using the Shapiro–Wilks test. We calculated Kaplan–Meier product limits for cumulative probability of reaching an endpoint and used the log-rank test for evidence of a statistically significant difference between the groups. Time was measured from the first admission for a procedure to outcomes. Cox regression analysis was used to estimate hazard ratios for the effect of thrombus aspiration in age-adjusted and fully adjusted models, based on covariates statistically different among the groups (Tables 1, 2). A propensity score analysis was carried out using a non parsimonious logistic regression model comparing TA and PPCI-only patients. The C-score was 0.78, indicating good discrimination. After ranking propensity score in an ascending order, a nearest neighbour 1:1 matching algorithm was used with callipers of 0.2 SD of the logit of the propensity score. Each TA and PPCI only patient was used in at most 1 matched pair to create a matched sample with similar distribution of baseline characteristics between observed groups. Based on the matched samples, the Cox proportional hazard model was used to determine the impact of TA on mortality over follow-up. All calculations were performed using the computer program SPSS 12.

**Table 1:** Baseline and follow-up clinical characteristics, angiographic and procedural data in overall study-population

	TA group		P	No TA group		P
	Basal	Follow-up		Basal	Follow-up	
N	1000	1000		1504	1504	
Mean age (years)	68.1 ± 6.5	–		67.2 ± 6.4*	–	
BMI (kg/m^2^)	27.0 ± 2.0	26.9 ± 1.9	0.001	27.6 ± 1.8*	26.9 ± 2.1	0.001
SBP (mmHg)	128.0 ± 13.0	123.0 ± 11.7	0.001	127.0 ± 13.1	125.0 ± 13.1*	0.001
DBP (mmHg)	79.0 ± 6.8	76.0 ± 6.6	0.001	79.0 ± 6.7	76.2 ± 6.8	0.001
Heart rate (bpm)	85.0 ± 8.9	76.1 ± 8.6	0.001	87.0 ± 8.7*	77.0 ± 8.5*	0.001
TIMI flow grade, n (%)						
Grade 0	705 (70.5)	–	–	978 (65.0)*	–	–
Grade 1	157 (15.7)	–	–	218 (14.5)	–	–
Grade 2/3	92 (13.8)	–	–	309 (20.5)*	–	–
Killip class 3–4, n (%)	60 (6)	–		76 (5)	–	
Diabetes, n (%)	185 (18.5)	–	–	283 (18.8)	–	–
Newly diabetes, n (%)	–	136 (13.6)		–	228 (15.2)	–
Hypertension, n (%)	503 (50.3)	–	–	743 (49.4)	–	–
Hyperlipemia, n (%)	275 (27.5)	–	–	443 (29.5)	–	–
Cigarette smoking, n (%)	444 (44.4)	–	–	565 (37.6)*	–	–
Active treatments						
β-blockers, n (%)	296 (26.6)	824 (82.4)	0.001	392 (26.1)*	1221 (81.2)	0.001
ACE inhibitors, n (%)	211 (21.1)	504 (50.4)	0.001	272 (18.1)*	734 (48.8)	0.001
Angiotensin receptor blockers, n (%)	165 (16.5)	372 (37.2)	0.001	218 (14.5)	554 (36.8)	0.001
Calcium inhibitor, n (%)	129 (12.9)	210 (21.0)	0.001	159 (10.6)*	300 (19.9)	0.001
Nitrate, n (%)	–	577 (57.7)	–	–	916 (60.9)	
Statins, n (%)	286 (28.6)	917 (91.7)	0.001	428 (28.5)	1381 (91.8)	0.001
Diuretic, n (%)	96 (9.6)	92 (9.2)	0.409	175 (11.6)	111 (7.4)	0.001
Insulin, n (%)	78 (7.8)	174 (17.4)	0.001	129 (8.6)	353 (23.5)*	0.001
Oral anti-diabetic, n (%)	168 (16.8)	282 (28.2)	0.001	266 (17.6)	534 (35.5)*	0.001
Aspirin, n (%)	432 (43.2)	973 (97.3)	0.001	601 (40.0)	1459 (97.0)*	0.001
Thienopyridine, n (%)	63 (6.3)	949 (94.9)	0.001	120 (8.0)	1432 (95.2)	0.001
Dual anti-aggregant therapy	–	937 (93.7)	–	–	1399 (93.0)	–
Low-molecular heparin, n (%)	–	12 (12.4)	–	–	190 (12.6)	–
Vitamin-K antagonist, n (%)	–	37 (3.7)	–	–	54 (3.6)	–
Laboratory analyses						
Plasma glucose (mg/dl)	138.3 ± 26.4	109.1 ± 22.9	0.001	141.7 ± 50.1	109.6 ± 18.8	0.001
Cholesterol (mg/dl)	198.8 ± 20.3	194.4 ± 22.8	0.001	196.2 ± 24.1*	193.9 ± 23.6	0.01
LDL-cholesterol (mg/dl)	125.6 ± 19.6	121.2 ± 22.4	0.001	123.7 ± 22.7*	120.9 ± 22.2	0.001
HDL-cholesterol (mg/dl)	39.7 ± 3.6	41.1 ± 3.8	0.001	38.0 ± 3.7*	41.0 ± 3.9*	0.001
Triglycerides (mg/dl)	168.0 ± 24.0	160.3 ± 24.7	0.001	180.6 ± 24.4*	160.9 ± 22.2	0.001
Creatinine (mg/dl)	0.9 ± 0.2	1.0 ± 0.1	0.001	0.9 ± 0.1	1.01 ± 0.1	0.001
cTnT (ng/l)	5.6 ± 1.4	–	–	5.8 ± 1.7	–	–
Procedural data						
Symptom to angiography (min)	136.9 ± 37.2	–	–	135.8 ± 41.1	–	–
Insulin infusion time (min)	–	–	–	–	–	–
Angiographic data						
Number of diseased vessels, n (%)						
1-VD	704 (70.4)	–	–	1055 (70.1)	–	–
2-VD	244 (24.4)	–	–	356 (23.7)	–	–
3-VD	52 (5.2)	–	–	93 (6.2)	–	–
Lesion location, n (%)						
RCA	512 (51.2)	–	–	665 (44.2)*	–	–
LAD	390 (39.0)	–	–	581 (38.6)	–	–
LM	151 (15.1)	–	–	196 (13.0)	–	–
LCx	298 (29.8)	–	–	604 (40.2)*	–	–
Trombus grade—Sianos et al. n (%)						
G0 none	94 (9.4)	–	–	143 (9.5)	–	–
G1 possible	86 (12.9)	–	–	194 (12.9)	–	–
G2 small	78 (7.8)	–	–	124 (8.2)	–	–
G3 medium	181 (18.1)	–	–	275 (18.3)	–	–
G4 large	193 (19.3)	–	–	292 (19.4)	–	–
G5 v. occlusion	332 (33.2)	–	–	476 (31.6)	–	–
Dimension (largest) (mm)	5.5 ± 1.6	–	–	–	–	–
Dimension (length) (mm)	22.5 ± 1.9	–	–	–	–	–
LVEF, n (%)						
> 50	513 (51.3)	571 (57.1)	0.005	813 (54.1)	881 (58.5)	0.008
41–50	413 (41.3)	374 (37.4)	0.041	614 (40.8)	548 (36.8)	0.007
25–40	74 (7.4)	54 (5.4)	0.046	76 (5.1)*	75 (5.0)	0.533
Quantitative angiographic data						
Lesion length (mm)	20.5 ± 2.0	–	–	20.3 ± 2.0*	–	–
Reference diameter (mm)	2.7 ± 0.3	–	–	2.8 ± 0.2*	–	–
MLD (mm)	1.05 ± 0.2	–	–	1.07 ± 0.2*	–	–
MLD post (in-stent) (mm)	2.6 ± 0.3	–	–	2.6 ± 0.3	–	–
Stent types, n (%)						
BMS	455 (45.5)	–	–	642 (41.6)	–	–
DES	545 (54.5)	–	–	873 (56.4)	–	–
TIMI gr 3 post PCI	920 (92.0)	–	–	1355 (90.1)	–	–
TIMI gr 2 post PCI	54 (5.4)	–	–	92 (6.1)	–	–
TIMI gr 1 post PCI	22 (2.2)	–	–	47 (3.1)	–	–
TIMI gr 0 post PCI	4 (0.4)	–	–	12 (0.8)	–	–
Multivessel intervention	241 (24.1)	–	–	363 (24.1)	–	–
Clinical outcomes						
All cause deaths	–	56 (5.6)	–	–	96 (6.4)	–
Cardiac deaths	–	48 (4.8)	–	–	89 (5.9)	–
Acute coronary syndrome	–	67 (6.7)	–	–	107 (7.1)	–
Heart failure	–	121 (12.1)	–	–	186 (12.4)	–
Stroke	–	12 (1.2)	–	–	19 (1.3)	–

Data are mean ± SD or n (%)

*TA* thrombus-aspiration, *SBP* systolic blood pressure, *DBP* diastolic blood pressure, *LVEF* left ventricular ejection fraction, *RCA* right coronary artery, *LM* left main, *LAD* left anterior descending, *LCx* left circumflex artery, *MLD* minimum luminal diameter, *1-VD* single-vessel disease, *2-VD* two-vessel disease, *3-VD* three-vessel disease, *BMS* bare metal stent, *DES* drug-eluting stent, *TNF-α* tumor necrosis factor-alpha, *MMP-9* matrix metallopeptidase-9, *MDL* minimal lumen diameter, *PS* propensity score, *CD68* macrophages, *TNF-α* tumor necrosis factor-α

* P < 0.05 vs. TA patients

**Table 2:** Baseline and follow-up clinical characteristics, angiographic and procedural data in sub-group hyperglycemic patients

	TA group		P	No TA group		P
	Basal	Follow-up		Basal	Follow-up	
N	331	331		566	566	
Mean age (years)	68.5 ± 6.3	–		66.8 ± 6.4*	–	
BMI (kg/m^2^)	27.9 ± 1.7	26.7 ± 1.9	0.001	27.8 ± 1.6	26.9 ± 2.1*	0.001
SBP (mmHg)	123.7 ± 11.2	122.1 ± 11.7	0.001	126.7 ± 12.6*	126.1 ± 13.5*	0.077
DBP (mmHg)	79.1 ± 6.5	76.6 ± 6.6	0.001	78.8 ± 6.7	75.9 ± 6.7	0.001
Heart rate (bpm)	87.1 ± 8.0	77.1 ± 9.3	0.001	87.8 ± 8.4	76.7 ± 8.5	0.001
TIMI flow grade post PCI, n (%)						
Grade 0	13 (3.9)	–		43 (7.6)*	–	
Grade 1	81 (24.5)	–		191 (33.7)*	–	
Grade 2/3	237 (71.6)	–		332 (58.6)*	–	
Killip class 3–4, n (%)	24 (7)	–		34 (6)	–	
Diabetes, n (%)	67 (20.2)	–		116 (20.5)	–	
Newly diabetes, n (%)	–	32 (9.7)	–	–	50 (8.8)	
Hypertension, n (%)	129 (38.9)	–		220 (38.9)	–	
Hyperlipemia, n (%)	93 (28.1)	–		115 (27.4)	–	
Cigarette smoking, n (%)	132 (39.9)	–		178 (31.5)*	–	
Active treatments						
β-blockers, n (%)	91 (27.5)	260 (78.5)	0.001	155 (27.4)	442 (78.1)	0.001
ACE inhibitors, n (%)	59 (17.8)	140 (45.0)	0.001	105 (18.5)	257 (45.4)	0.001
Angiotensin receptor blockers, n (%)	45 (13.6)	114 (34.4)	0.001	83 (14.7)	201 (35.5)	0.001
Calcium inhibitor, n (%)	29 (8.8)	63 (19.0)	0.001	56 (9.9)	109 (19.3)	0.001
Nitrate, n (%)	–	195 (58.9)		–	320 (56.5)	
Statins, n (%)	83 (25.1)	308 (93.0)	0.001	143 (25.3)	526 (92.9)	0.001
Diuretic, n (%)	29 (8.8)	32 (9.7)	0.05	48 (8.5)	43 (7.6)	
Insulin, n (%)	35 (10.6)	48 (14.5)	0.01	60 (10.6)	65 (11.5)	0.05
Oral antidiabetic, n (%)	59 (17.8)	89 (26.9)	0.001	101 (17.8)	132 (23.3)	0.01
Aspirin, n (%)	146 (44.1)	327 (98.6)	0.001	251 (44.3)	557 (98.5)	0.001
Thienopyridine, n (%)	27 (8.2)	318 (96.1)	0.001	46 (8.1)	538 (95.0)	0.001
Dual anti-aggregant therapy	–	313 (94.6)	–	–	427 (93.1)	–
Low-molecular heparin, n (%)	–	41 (12.4)		–	76 (13.4)	–
Vitamin-K antagonist, n (%)	–	17 (5.1)		–	28 (4.9)	–
Laboratory analyses						
Plasma glucose (mg/dl)	201.4 ± 25.1	109.1 ± 23.5	0.001	200.1 ± 29.9	106.1 ± 22.5	0.001
Cholesterol (mg/dl)	201.9 ± 21.8	195.6 ± 22.1	0.001	198.1 ± 21.9*	194.7 ± 22.7	0.001
LDL-cholesterol (mg/dl)	128.3 ± 21.4	122.4 ± 22.1	0.001	124.3 ± 18.6*	121.7 ± 22.3	0.001
HDL-cholesterol (mg/dl)	37.3 ± 3.4	41.2 ± 3.6	0.001	38.0 ± 3.5*	40.7 ± 3.9	0.001
Triglycerides (mg/dl)	185.3 ± 23.5	160.1 ± 31.9	0.001	185.7 ± 22.5	161.5 ± 22.7	0.001
Creatinine (mg/dl)	0.99 ± 0.15	1.04 ± 0.13	0.001	0.99 ± 0.14	1.4 ± 0.14	0.001
cTnT (ng/l)	5.7 ± 2.1	–	–	5.9 ± 1.8	–	–
Procedural data						
Symptom to angiography (min)	133.8 ± 38.9	–	–	135.1 ± 50.7	–	–
Insulin infusion time (min)	37.7 ± 5.2	–	–	37.3 ± 4.4	–	–
Angiographic data						
Number of diseased vessels, n (%)						
1-VD	228 (68.9)	–	–	388 (68.5)	–	–
2-VD	86 (26.0)	–	–	143 (25.3)	–	–
3-VD	17 (5.1)	–	–	35 (6.2)	–	–
Lesion location, n (%)						
RCA	185 (55.9)	–	–	257 (45.4)*	–	–
LAD	118 (36.5)	–	–	228 (40.3)	–	–
LM	45 (13.6)	–	–	79 (13.9)	–	–
LCx	103 (31.1)	–	–	215 (39.9)*	–	–
Thrombus grade—Sianos et al., n (%)						
G0 none	30 (9.1)	–	–	62 (10.9)	–	–
G1 possible	38 (11.5)	–	–	70 (12.4)	–	–
G2 small	16 (4.8)	–	–	35 (6.2)	–	–
G3 medium	57 (17.2)	–	–	99 (17.5)	–	–
G4 large	62 (18.7)	–	–	108 (19.1)	–	–
G5 v. occlusion	128 (38.7)	–	–	192 (33.9)	–	–
Thrombus size (mm)	34 ± 1.9	–	–	–	–	–
LVEF, n (%)						
> 50	165 (49.9)	198 (59.8)	0.006	288 (50.9)	331 (58.5)	0.006
41–50	151 (45.6)	116 (35.0)	0.004	249 (44.0)	208 (36.7)	0.01
25–40	15 (4.5)	17 (5.1)	0.428	29 (5.1)	27 (4.8)	0.139
Quantitative angiographic data						
Lesion length (mm)	20.3 ± 2.0	–	–	20.2 ± 2.1	–	–
Reference diameter (mm)	2.7 ± 0.4	–	–	2.8 ± 0.3	–	–
MLD (Mm)	1.05 ± 0.29	–	–	1.07 ± 0.16	–	–
MLD post (in-stent) (mm)	2.7 ± 0.3	–	–	2.7 ± 0.2	–	–
Stent types, n (%)						
BMS	146 (44.1)	–	–	233 (41.2)	–	–
DES	185 (55.9)	–	–	333 (58.8)	–	–
TIMI gr 3 post PCI	285 (86.1)	–	–	498 (87.9)	–	–
Multivessel intervention	79 (23.9)	–	–	134 (23.7)	–	–
Clinical outcomes						
All cause deaths	–	27 (8.2)		–	74 (13.1)	–
Cardiac deaths	–	17 (5.1)		–	62 (10.9)	–
Acute coronary syndrome	–	25 (7.5)		–	67 (11.8)	–
Heart failure	–	53 (16.1)		–	93 (16.4)	–
Stroke	–	6 (1.8)		–	11 (1.9)	–

Data are mean ± SD or n (%)

*TA* thrombus-aspiration, *SBP* systolic blood pressure, *DBP* diastolic blood pressure, *LVEF* left ventricular ejection fraction, *RCA* right coronary artery, *LM* left main, *LAD* left anterior descending, *LCx* left circumflex artery, *MLD* minimum luminal diameter, *1-VD* single-vessel disease, *2-VD* two-vessel disease, *3-VD* three-vessel disease, *BMS* bare metal stent, *DES* drug-eluting stent, *TNF-α* tumor necrosis factor-alpha, *MMP-9* matrix metallopeptidase-9, *MDL* minimal lumen diameter, *PS* propensity score, *CD68* macrophages, *TNF-α* tumor necrosis factor-α

* P < 0.05 vs. TA patients

## Results

### Patient characteristics

**Fig. 1:**
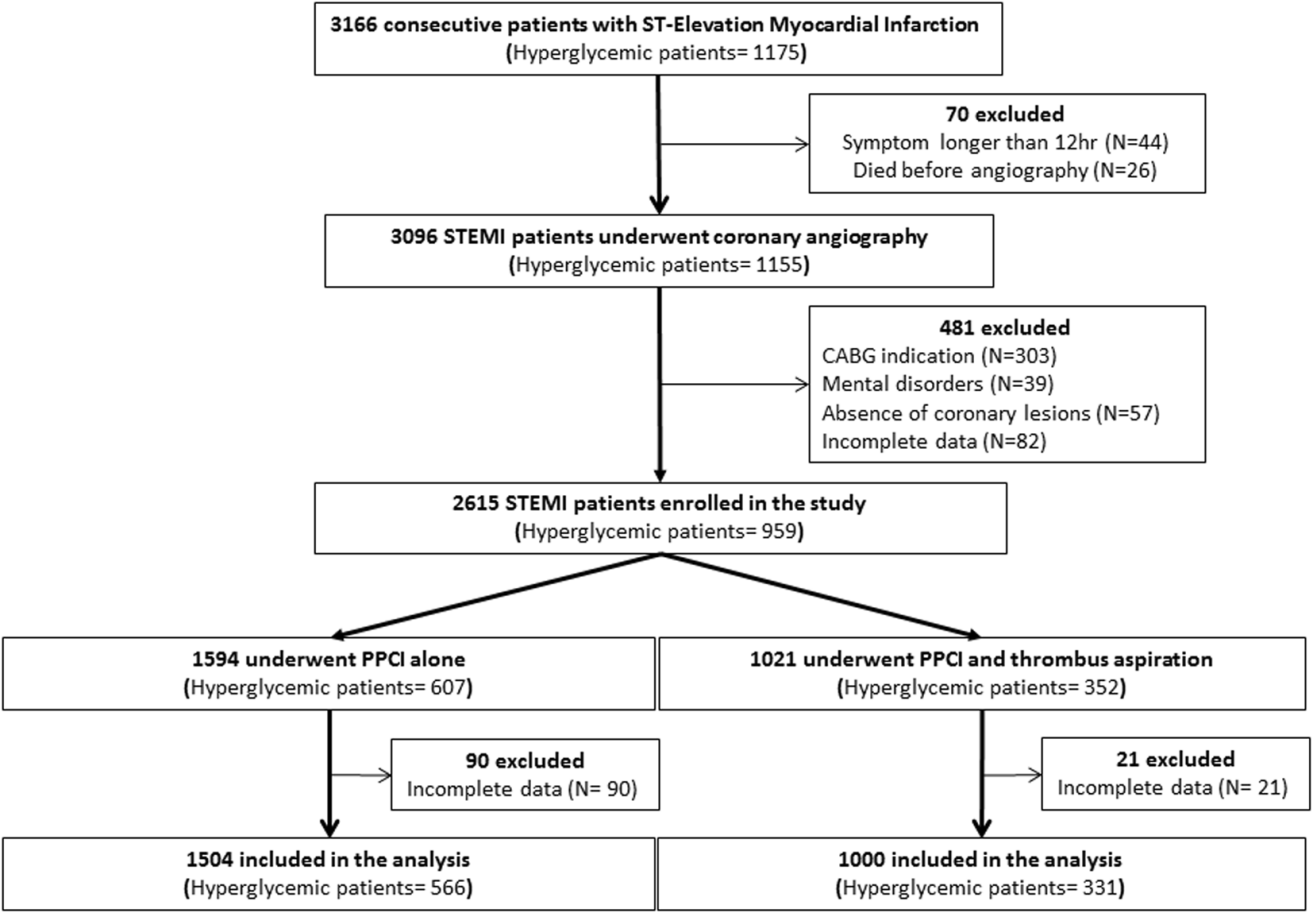
Study flow diagram

Three thousand and sixty six patients with first STEMI (1175 with hyperglycaemia) were admitted at cardiologic study centres between February 2008 and July 2015. Of these 44 patients have symptoms longer than 12 h and 26 died before PPCI, thus 70 patients were excluded from the evaluation. Consequently, 3096 eligible STEMI patients underwent to PPCI (1155 with hyperglycaemia). After coronary study, 303 patients were excluded for CABG indication, 39 for mental disorders, 57 for absence of coronary lesions and 82 for incomplete data. Thus, 2615 patients were enrolled in the study (959 with hyperglycaemia) (Fig. 1). Of these patients, 1594 were treated with PPCI alone (607 with hyperglycaemia) and 1021 with PPCI plus TA (352 with hyperglycaemia). During the follow-up, 90 patients in no-TA group and 21 patients in TA group withdraw from the study voluntarily and/or were excluded for incomplete data. Therefore, the final study cohort included 1504 no-TA patients (566 with hyperglycaemia) and 1000 TA patients (331 with hyperglycaemia) followed for 1 year. In overall population as well as in the hyperglycemic sub-group, patients in the TA group were older, had poor pre-procedure coronary artery alteration compared with the PPCI-only group (Tables 1, 2). Moreover, TA patients had higher plasmacholesterol, low-density lipoprotein (LDL), and lower high-density lipoprotein (HDL) levels (Tables 1, 2). No differences in the troponin levels, in the median time between symptom onset and the start of angiography procedure at hemodynamic unit, in the number of coronary vessel diseases, thrombus grade and in the number of patients with post-procedural TIMI 3 were observed (Tables 1, 2). Finally, the culprit lesion was more frequently located in the right coronary artery (RCA) in TA patients, and in the left circumflex artery (LM) in patients treated with conventional PPCI.

### In-hospital treatments and glucose control

During hospital stay, hyperglycemic patients were treated according to international guidelines [23], with high proportions of patients receiving platelet inhibitors and antithrombotic agents before, during and after the procedure. In particular, aspirin was administered to 96% of patients and statins to 89% in both groups. Beta-blockers were given to 85% of patients and 40% received an ACE-inhibitor in both groups. Among whole population, 86% of the patients were treated with association between thienopyridine and aspirin: 85% of TA patients and 87% of no-TA patients. In the sub-group of hyperglycemic patients, the mean plasma glucose level during the peri-angiographic period was similar in the groups (TA group, 201.1 ± 25 vs. no-TA group, 201.4 ± 29 mg/dl). After PPCI, glycemic goal was maintained for 24 h in both groups (TA group, 166 ± 11 mg/dl; no-TA group, 165 ± 14; mg/dl). Blood glucose < 70 mg/dl with and without symptoms occurred during the insulin infusion in the 10% of TA patients and in 11% of no-TA patients. At hospital discharge, both fasting and post-prandial plasma glucose levels were similar in the groups (TA-group, fasting, 139 ± 29 mg/dl, post-prandial 181 ± 16 mg/dl; no-TA group, 136 ± 30 mg/dl, post-prandial 181 ± 23 mg/dl). At discharge from hospital, the rate of the use of ACE-inhibitors, aspirin, beta-blockers, statins, and thienopyridines increased compared to their use at admission in both TA and no-TA population (Tables 1 and 2).

### Treatments at 12 months following discharge

Between presentation of STEMI and the 12 months following hospital discharge, 364 patients (14.5%) in the entire study cohort had new evidence of diabetes (Table 1). Among the hyperglycemic sub-group patients, 32 (9.7%) in the TA group and 50 (8.8%) in the no-TA group cohort had new evidence of diabetes (Table 2). Diabetic patients evidenced no differences in metabolic control between the groups in overall population and in hyperglycemic sub-group (Tables 1, 2). Over the 1 year follow-up period, there was no difference in both anti-diabetic and cardiovascular therapies during the follow up among the groups (Tables 1, 2).

### In-hospital and post-discharge outcomes

**Fig. 2:**
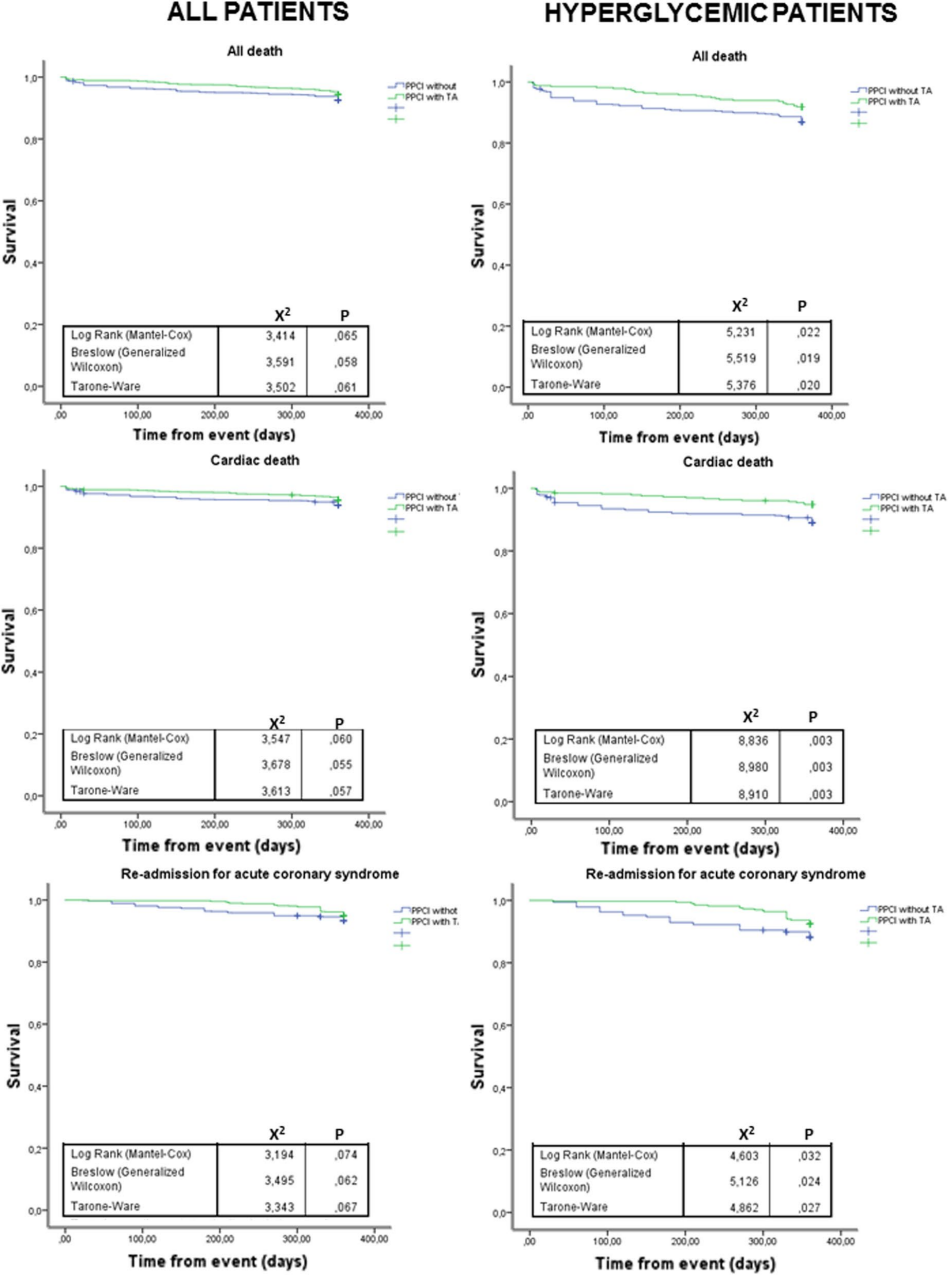
Kaplan–Meier curves showing cumulative incidence of readmission and mortality from 1 year after hospital discharge stratified by thrombus aspiration (with TA) and no thrombus aspiration (without TA) in all population and hyperglycemic sub-group patients

**Fig. 3:**
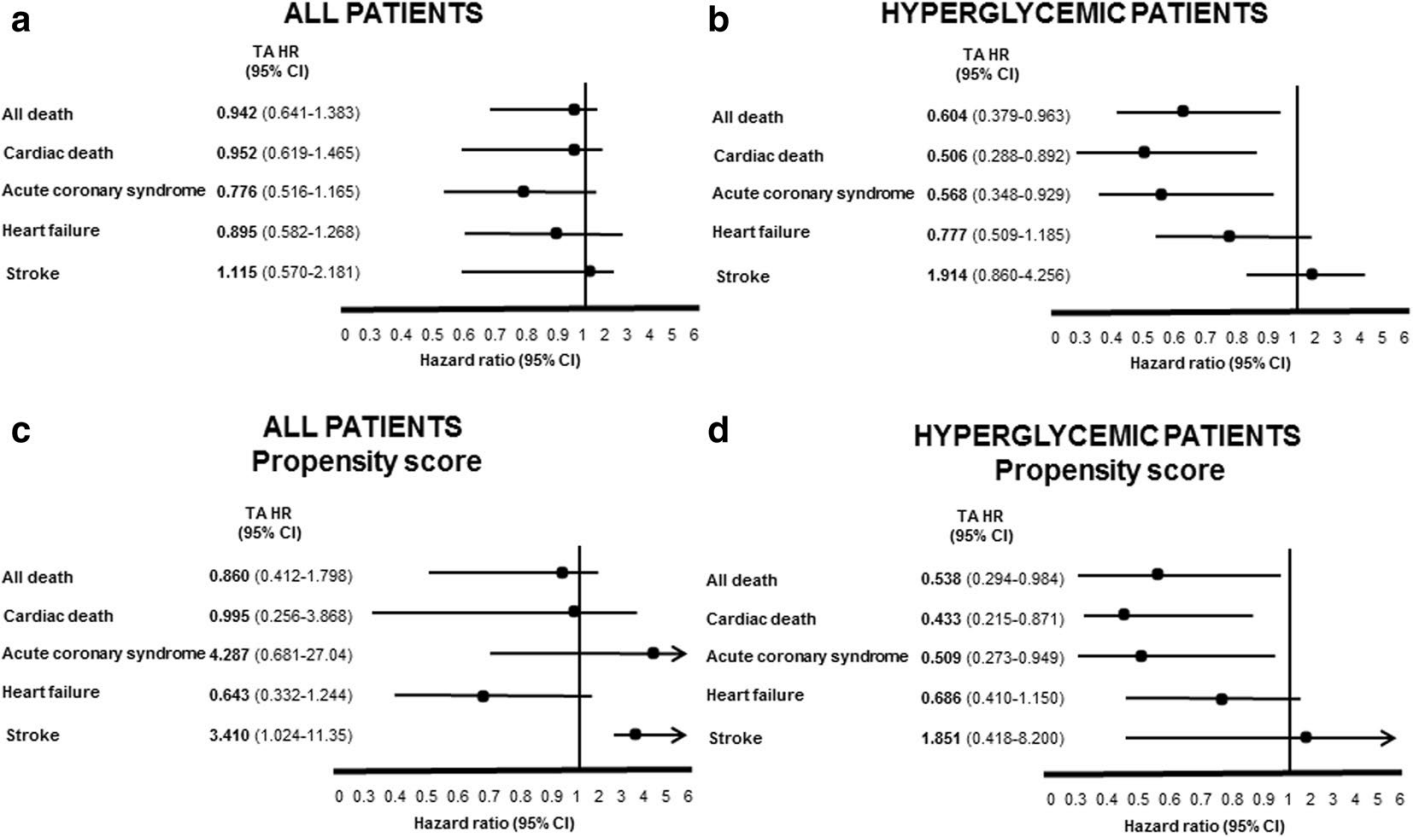
Hazard ratios (HR) and associated 95% confidence intervals are shown for all death, cardiac death, re-admission for acute coronary syndrome, heart failure and stroke adjusted for age, BMI, heart rate, systolic blood pressure, total cholesterol, HDL and LDL-cholesterol levels, triglycerides, TIMI scores, lesions of RCA and LM, and ejection fraction at baseline (full cohort and propensity matched cohort) in all population and hyperglycemic sub-group patients. The black circle indicates HR, and horizontal lines indicate 95% confidence intervals. TA, thrombus aspiration

Of the studied patients who were enrolled in the study, 1.1% died while in hospital. The in-hospital mortality rate did not differ between patients treated with or without TA. In the overall population, after 12 months following PPCI, the unadjusted Kaplan–Meier estimated survival curve showed no difference in all and cardiac mortality in patients who received TA compared with those who did not (Fig. 2). Moreover, there were no differences between groups in the rate of re-admission for SCA (P = 0.74), HF (P = 0.18) or incident stroke (P = 0.67). Moreover, multivariate-adjusted Cox analysis, including covariates statistically different among the groups, revealed that the use of TA in PPCI was not associated with all-cause mortality (HR: 0.942, 95% CI 0.641–1.383), cardiac death (HR: 0.952, 95% CI 0.619–1.465), SCA (HR: 0.776, 95% CI 0.516–1.165) (Fig. 3a), HF (HR: 0.895, 95% CI 0.582–1.268) and stroke (HR: 1.115, 95% CI 0.570–2.181). To further account for confounding variables and bias, propensity score matching was performed to adjust for differences in demographic and procedural variables producing a total of 1994 patients (997 in the TA group and 997 in the no-TA group). Following matching, the baseline demographics and procedural variables were well balanced in the two PS-matched cohorts. In the PS-matched cohorts, Cox regression analysis revealed that the use of TA during PPCI was not a predictor of all-cause mortality (HR: 0.860, 95% CI 0.412–1.798), cardiac death (HR: 0.995, 95% CI 0.256–3.868), SCA (HR: 4.287, 95% CI 0.681–27.04) and HF (HR: 0.643, 95% CI 0.332–1.244), but was associated to an increase in stroke rate (HR: 3.410, 95% CI 1.024–11.35), (Fig. 3c). Conversely in the sub-group of hyperglycemic patients, the unadjusted Kaplan–Meier showed that all death rate at 1 year was 8.2% in TA group v/s. 13.1% in no-TA group (P < 0.019) (Fig. 2). Furthermore, we categorized causes of death into cardiovascular death and not cardiac. The point estimates indicated a stronger association between both no-TA and TA patients and cardiovascular death than non-cardiovascular death. The cardiac death rate at 1 year was 10.9% in patients with no-TA patients vs. 5.1% in TA patients (P < 0.003) (Fig. 2, Tables 1, 2). The incidence of readmission for SCA through 12 months was distributed in a fashion similar to that of mortality rates across the 2 groups (Fig. 2, Tables 1, 2). Following discharge from hospital, 7.5% of TA patients and 11.8% ofno-TA patients were re-hospitalized for coronary diseases (P < 0.024). The incidence of readmission for heart failure at 12-months was no increased in TA patients compared to no-TA patients (P = 0.247). The incidence of readmission for stroke at 12-months was no increased in TA patients compared to no-TA patients (P = 0.875). The outcomes were also analyzed with Cox regression analysis by covariates statistically different among the groups. After the adjustments for age, BMI, systolic blood pressure, total cholesterol, HDL and LDL-cholesterol levels, triglycerides, TIMI scores, lesions of RCA and LM, and ejection fraction, adjusted Cox regression analysis showed significantly higher 1-year overall survival for all-cause mortality (HR: 0.604, 95% CI 0.379–0.963), cardiac death (HR: 0.506, 95% CI 0.288–0.892) and SCA (HR: 0.568, 95% CI 0.348–929) in patients treated with TA. However, after risks adjustment, HF and stroke at 1 year were similar between the groups (P = 0.171) (Fig. 3b). To further account for confounding variables and bias, propensity score matching was performed to adjust for differences in demographic and procedural variables producing a total of 546 patients (273 in the TA group and 273 in the no-TA group). Following matching, the baseline demographics and procedural variables were well balanced in the two propensity-matched cohorts. Cox regression analysis, revealed that the use of TA during PPCI was associated with 46% reduction of all-cause mortality (HR: 0.538, 95% CI 0.294–0.984), 57% of cardiac death (HR: 0.433, 95% CI 0.215–0.871) and 49% of re-admission for SCA (HR: 0.509, 95% CI 0.273–0.949). Finally, Cox regression analysis revealed that the use of TA during PPCI was not associated with a reduction risk of re-admission for HF (HR: 0.686, 95% CI 0.410–1.150) and stroke (HR: 1.851, 95% CI 0.418–8.200) (Fig. 3d).

### Coronary thrombus analysis in hyperglycemic and normo-glycemic patients

**Fig. 4:**
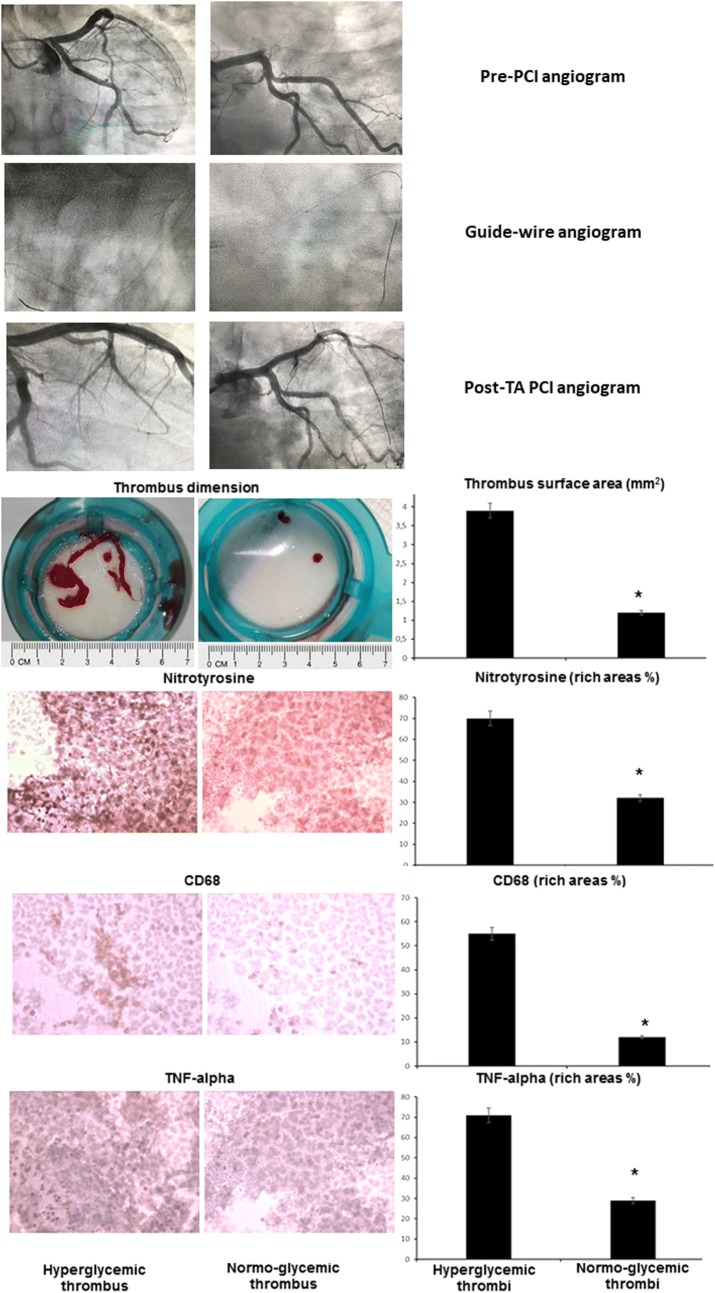
Representation of angiographic data pre and post thrombus aspiration (TA) and percutaneous coronary intervention (PCI) in the upper part of the figure. In the middle part of the figure the thrombus dimension as surface area, and in the lower part of the figure the immunohistochemistry images of the serial sections incubated with the anti-CD68, tumor necrosis factor-α (TNF-α), and nitrotyrosine comparing hyperglycemic vs. normo-glycemic thrombus (*P value < 0.05). In the right inferior part of the figure, the bar graphs representing areas with higher vs. lower expression of the analyzed marker, that are expressed in percentage, and called as rich areas %. Hyperglycemics vs. normoglycemics, *P value < 0.05

The volume of the thrombi, the number of retrieved fragments, and the size of the thrombi were significantly higher in hyperglycemic compared to normo-glycemic patients. Thrombi composition evidenced higher leukocytes, and lower fibrin content in hyperglycemic compared to normo-glycemic patients (Fig. 4, Table 3). Moreover, immunohistochemistry and ELISA analysis revealed markedly higher staining and levels of macrophages (CD68), TNF-α, MMP-9 and nitrotyrosine in all hyperglycemic vs. normoglycemic thrombi (P < 0.001, for all) (Fig. 4 Table 3).

**Table 3:** Histopathological characteristics of coronary-artery thrombi from initial findings on coronary angiography in 50 hyperglycemic and 50 normo-glycemic PS-matched patients undergoing thrombus aspiration

Characteristics of the thrombus	Hyperglycemic patients	Normo-glycemic patients	P
Dimension (largest) (mm)	8.9 ± 2.7	2.2 ± 1.2*	0.001
Dimension (length) (mm)	34 ± 2.9	11 ± 0.8	
Fibrin (%)	38.6 ± 11.1	62.4 ± 15.6*	0.001
Platelet (%)	11.3 ± 6.2	12.5 ± 3.2	0.001
CD68 (%)	24.7 ± 4.9	9.1 ± 3.2*	0.001
TNF-α (pg/mg)	44.7 ± 12.1	19.3 ± 10.5*	0.001
MMP-9 (µg/mg)	16.9 ± 4.4	6.2 ± 1.6*	0.001
Nitrotyrosine (nmol/pg)	2.5 ± 0.21	1.1 ± 0.24	0.001

*statisticaly significant (P value <0.05)

## Discussion

### TA in general STEMI population outcomes

The role of coronary TA in PPCI for STEMI has been controversial. An appealing biological rationale, coupled with encouraging early trial results, fueled its uptake over the early part of the last decade. Now, in the wake of large RCTs [12–15] and recent meta-analysis study on 19.000 patients that failed to show any important clinical benefits [24], its place in our therapeutic armamentarium is unclear in general population. According to these evidences, our data suggest that in-hospital mortality and event survival were similar for the patients who underwent TA to those who received PCI only, suggesting that, in our overall cohort, routine TA during PPCI, compared with not receiving therapy, does not appear to be associated with a reduction in mortality and event rates in general population. Very different, however, are the data related to STEMI population with hyperglycemia (blood glucose levels > 140 mg/dl) at admission. In our cohort of consecutive patients with first STEMI treated with PPCI, 897 patients were hyperglycemic at admission (36% of total population), and 331 of patients (36.9%) underwent TA during PPCI. In patients who underwent TA as compared to those who received PPCI only, we observed lower mortality and higher event survival. In the unadjusted analysis of the hyperglycemic cohort, 8.1% of patients treated with adjunctive TA died within 1 year, as compared with 13.1% of patients treated with conventional PPCI (all cause deaths). Thus, beyond early restoration of epicardial blood flow, TA during PPCI, limiting distal embolization and preserving microcirculatory integrity [25], may have a central role in the management of hyperglycemic STEMI patients. Accordingly, the observation, that the rate of post-PCI TIMI flow grades 2/3 observed in our study among hyperglycemic patients undergoing primary PCI plus TA is high as that reported after primary PCI without TA, could underline the importance of thrombus-aspiration in a group of high-risk patients such as patients with hyperglycemia during STEMI in preserving microvascular perfusion.

### TA in hyperglycemic STEMI population outcomes

Despite its strong data and intuitive basis for incremental value of TA in hyperglycemic patients, results from pivotal trials and “real-world” registries of manual thrombectomy have not consistently demonstrated clinical benefits over standard PPCI in general population [20, 26, 27]. However, all these studies did not provide any evidence about the effects of TA in a subgroup of high-risk STEMI patients, such as those with hyperglycemia during the event. In this context, in a contemporary sample of hyperglycemic patients with STEMI treated with PPCI in routine practice, we observed higher cumulative incidence of 1-year mortality and adverse cardiovascular outcomes, compared to hyperglycemic patients with STEMI treated with revascularization strategy and TA. Moreover, the 1-year prognosis of hyperglycemic patients with STEMI without TA was significantly worse, with 29.1% a rate of re-admission for cardiovascular diseases, than hyperglycemic patients with STEMI plus TA (22.6% a rate of re-admission for cardiovascular diseases) at follow-up. In our Tahiti study after STEMI, the 1-year follow-up results show a 49% of reduction in the primary endpoint of all death and 55% of cardiac death by TA in STEMI compared with no TA during PPCI in STEMI patients despite a similar severity of atherosclerotic disease at baseline and similar glycemic control at baseline and at follow-up. The number of hyperglycemic patients in this report was large, and the samples of patients with STEMI treated with conventional PPCI and STEMI patients treated with PPCI plus TA were population-based, suggesting that these results may be generalizable.

### Effects of TA in hyperglycemic STEMI patients: physio-pathological hypothesis

The reduction in mortality and cardiovascular events observed in hyperglycemic STEMI patients may be hypothesized to be the result of improved myocardial perfusion at tissue level. The TA may result in improved angiographic myocardial blush grade and better ST segment elevation resolution, which is both a marker for myocardial (re)perfusion at tissue level [17]. In the Bayesian meta-analysis, treatment with TA reduced the occurrence of distal embolization and microvascular obstruction [28]. Microvascular obstruction is associated with increased left ventricular remodeling, larger infarct size, the occurrence of ventricular arrhythmia and major adverse cardiac events [29]. These factors may contribute to mortality after myocardial infarction, as evidenced in our study. Nonetheless, the TA seems not counteract these physio-pathological mechanisms in general, as evidenced by our data and recent trials [20]. Moreover, it has been shown that morphologic and histopathologic constituency of coronary thrombi in the setting of a ST-elevation myocardial infarction was not significantly different between patients with or without diabetes [30, 31]. However, these aspects have not been analyzed in patients with STEMI and hyperglycemia. In this scenario, the hyperglycemic milieu may affect several pathogenetic mechanisms that favor the microvascular dysfunction, including inflammation, endothelial dysfunction with the inability to augment coronary flow in response to stress, and vasospasm [9]. Therefore, unlike the thrombus in normo-glycemia conditions, the presence of hyperglycemia could increase the production of oxidative stress and inflammation, responsible for the dysfunction of the microcirculation, from atherosclerotic plaque and thrombus. In line with such evidence, concomitantly higher levels of oxidative stress (nitrotyrosine levels) and inflammatory cytokines (TNF-α) as well as inflammatory cells were found in coronary thrombi obtained from the hyperglycemic STEMI patients compared with thrombi from normo-glycemic STEMI patients. Moreover, we observed that the size of the thrombi was significantly higher in hyperglycemic compared to normo-glycemic patients. In this context, the recent meta-analysis [24], while not showing any benefit of the TA on outcomes in patients with STEMI, shows that in the subgroup of patients with high thrombus burden, there was a nominal reduction in CV mortality and in all-cause mortality. Conversely, the TA in hyperglycemics may lead to identification of local inflammatory/oxidative pathways, affecting not only the intracoronary thrombus burden, but also the intracoronary thrombus inflammatory/oxidative activity. Therefore, more robust evidence would have needed also in thrombus composition, as pro-inflammatory burden of thrombi. In this setting, stress hyperglycemia during STEMI, may affects several pathogenetic mechanisms that influence coronary thrombus composition, including increase of inflammation [32, 33]. In the meantime, the question that arise is whether the mere quantitative evaluation of thrombus burden is sufficient to individualize subjects who can benefit from thrombus-aspiration in high risk patients as hyperglycemic-STEMI patients. In line with such evidences, the Tahiti study firstly suggests that TA before PPCI can theoretically protect the peri-infarct myocardium reducing the existing source of the excess of inflammation and oxidative stress resulting from an effect of hyperglycemia on the non-aspirated thrombus in conventional PPCI.

### Study limitations

Our analysis has several limitations. First, the study design was a non randomized observational cohort study. However, we used rigorous adjustments for confounding, such as PS matching. While these statistical methods were used to eliminate differences in observed confounders that affected a patients’ risk profile, they are unable to account for differences in unobserved confounders. A second limitation of our study is that we had no data on surrogate endpoints such as myocardial blush grade, ST-segment resolution, or infarct size. A third limitation is the high use of BMS in the study population that may influence the results [34, 35]. Nevertheless, there were no differences between hyperglycemic patients treated with BMS or DES (Additional file 1: Figure S1).

In conclusion, adjunctive TA was associated with a reduction in one-year mortality in hyperglycemic STEMI patients as compared with conventional PPCI. Thus, beyond early restoration of epicardial blood flow, TA during PPCI, limiting inflammatory distal embolization and preserving microcirculatory integrity, may have a central role in the management of hyperglycemic-STEMI patients. In the meantime, the question that arise is whether the mere quantitative evaluation of thrombus burden is sufficient to individualize subjects who can benefit from thrombus-aspiration in high risk patients as hyperglycemic-STEMI patients, as indicated by current practice. However, our observations merit confirmation in large randomized trials powered for detecting a difference in mortality of hyperglycemic STEMI patients.

## Additional file

**Additional file 1.** Kaplan Mayer curves in BMS and DES treated patients.

## Abbreviations

AH: admission hyperglycemia
AMI: acute myocardial infarction
PPCI: primary percutaneous coronary intervention
STEMI: ST-segment elevation myocardial infarction
TA: thrombus aspiration
PS: propensity score
MMP-9: matrix metallopeptidase-9
TNF-α: tumor necrosis factor-α
LDL: low-density lipoprotein
HDL: high-density lipoprotein
RCA: right coronary artery
LM: left circumflex artery
TAHITI: thrombus aspiration in hyperglycemic ST-elevation myocardial infarction (STEMI) patients

## References

[1] Capes SE, Hunt D, Malmberg K, Gerstein HC (2000). Stress hyperglycaemia and increased risk of death after myocardial infarction in patients with and without diabetes: a systematic overview. Lancet.

[2] Deedwania P, Kosiborod M, Barrett E, American Heart Association Diabetes Committee of the Council on Nutrition, Physical Activity, and Metabolism (2008). Hyperglycemia and acute coronary syndrome: a scientific statement from the American heart association diabetes committee of the council on nutrition, physical activity, and metabolism. Circulation.

[3] Singh K, Hibbert B, Singh B (2015). Meta-analysis of admission hyperglycemia in acute myocardial infarction patients treated with primary angioplasty: a cause or a marker of mortality?. Eur Heart J Cardiovasc Pharmacother.

[4] Marfella R, Sasso FC, Siniscalchi M (2012). Peri-procedural tight glycemic control during early percutaneous coronary intervention is associated with a lower rate of in-stent restenosis in patients with acute ST-elevation myocardial infarction. J Clin Endocrinol Metab.

[5] Ekmekci A, Cicek G, Uluganyan M (2014). Admission hyperglycemia predicts in hospital mortality and major adverse cardiac events after primary percutaneous coronary intervention in patients without diabetes mellitus. Angiology.

[6] Marfella R, Rizzo MR, Siniscalchi M (2013). Peri-procedural tight glycemic control during early percutaneous coronary intervention up-regulates endothelial progenitor cell level and differentiation during acute ST-elevation myocardial infarction: effects on myocardial salvage. Int J Cardiol.

[7] Marfella R, Siniscalchi M, Esposito K (2006). Effects of stress hyperglycemia on acute myocardial infarction: role of inflammatory immune process in functional cardiac outcome. Diabetes Care.

[8] Marfella R, Di Filippo C, Portoghese M (2009). Tight glycemic control reduces heart inflammation and remodeling during acute myocardial infarction in hyperglycemic patients. J Am Coll Cardiol.

[9] Marfella R, Paolisso G (2015). Glycemic control and acute coronary syndrome: the debate continues. Eur Heart J Cardiovasc Pharmacother.

[10] Birkhead J, Weston C, Timmis A, Chen R (2015). The effects of intravenous insulin infusions on early mortality for patients with acute coronary syndromes who present with hyperglycaemia: a matched propensity analysis using data from the MINAP database 2008–2012. Eur Heart J Acute Cardiovasc Care.

[11] Dandona P, Boden WE (2013). Intensive glucose control in hyperglycemic patients with acute coronary syndromes: still smoke, but no fire…. JAMA Intern Med.

[12] Malmberg K, DIGAMI (Diabetes Mellitus, Insulin Glucose Infusion in Acute Myocardial Infarction) Study Group (1997). Prospective randomised study of intensive insulin treatment on long term survival after acute myocardial infarction in patients with diabetes mellitus. BMJ.

[13] Mehta SR, Yusuf S, Díaz R, CREATE-ECLA Trial Group Investigators (2005). Effect of glucose-insulin-potassium infusion on mortality in patients with acute ST-segment elevation myocardial infarction: the CREATE-ECLA randomized controlled trial. JAMA.

[14] Selker HP, Beshansky JR, Sheehan PR (2012). Out-of-hospital administration of intravenous glucose-insulin-potassium in patients with suspected acute coronary syndromes: the IMMEDIATE randomized controlled trial. JAMA.

[15] Finfer S, Liu B, Chittock DR, Norton R, NICE-SUGAR Study Investigators (2012). Hypoglycemia and risk of death in critically ill patients. N Engl J Med.

[16] Radke PW, Schunkert H (2008). Glucose-lowering therapy after myocardial infarction: more questions than answers. Eur Heart J.

[17] Ikari Y, Sakurada M, Kozuma K, VAMPIRE Investigators (2008). Upfront thrombus aspiration in primary coronary intervention for patients with ST-segment elevation acute myocardial infarction: report of the VAMPIRE (VAcuuMasPIration thrombus REmoval) trial. JACC Cardiovasc Interv.

[18] Jolly SS, Cairns JA, Yusuf S, TOTAL Investigators (2016). Outcomes after thrombus aspiration for ST elevation myocardial infarction: 1-year follow-up of the prospective randomised TOTAL trial. Lancet.

[19] Lagerqvist B, Frobert O, Olivecrona GK (2014). Outcomes 1 year after thrombus aspiration for myocardial infarction. N Engl J Med.

[20] Tilsted HH, Olivecrona GK (2015). To aspirate or not to aspirate: that is the question. JACC Cardiovasc Interv.

[21] Paneni F, Beckman JA, Creager MA, Cosentino F (2013). Diabetes and vascular disease: pathophysiology, clinical consequences, and medical therapy: part I. Eur Heart J.

[22] Sianos G, Papafaklis MI, Serruys PW (2010). Angiographic thrombus burden classification in patients with ST-segment elevation myocardial infarction treated with percutaneous coronary intervention. J Invasive Cardiol.

[23] Kushner FG, Hand M, Smith SC, King SB, Anderson JL, Antman EM, Bailey SR, Bates ER, Blankenship JC, Casey DE, Green LA (2009). 2009 focused updates: ACC/AHA guidelines for the management of patients with ST-elevation myocardial infarction (updating the 2004 guideline and 2007 focused update) and ACC/AHA/SCAI guidelines on percutaneous coronary intervention (updating the 2005 guideline and 2007 focused update). J Am Coll Cardiol.

[24] Jolly SS, James S, Džavík V (2017). Thrombus aspiration in ST-segment-elevation myocardial infarction: an individual patient meta-analysis: Thrombectomy Trialists Collaboration. Circulation.

[25] Liistro F, Grotti S, Angioli P (2009). Impact of thrombus aspiration on myocardial tissue reperfusion and left ventricular functional recovery and remodeling after primary angioplasty. Circ Cardiovasc Interv.

[26] Vaduganathan M, Bhatt D (2015). Manual thrombectomy in myocardial infarction: aspiring for better. J Am Heart Assoc.

[27] Taglieri N, Bacchi Reggiani ML (2018). Efficacy and safety of thrombus aspiration in ST-segment elevation myocardial infarction: an updated systematic review and meta-analysis of randomised clinical trials. Eur Heart J Acute Cardiovasc Care.

[28] Mongeon FP, Belisle P, Joseph L, Eisenberg MJ, Rinfret S (2010). Adjunctive thrombectomy for acute myocardial infarction: a bayesian meta-analysis. Circ Cardiovasc Interv.

[29] Gerber BL, Rochitte CE, Melin JA (2000). Microvascular obstruction and left ventricular remodeling early after acute myocardial infarction. Circulation.

[30] Sebben JC, Pinto Ribeiro DR, Lopes RD (2016). The role of diabetes mellitus in the composition of coronary thrombi in patients presenting with acute ST-segment elevation myocardial infarction undergoing primary percutaneous coronary intervention. Am Heart J.

[31] Li X, Kramer MC, Damman P, van der Wal AC (2016). Older coronary thrombus is an independent predictor of 1-year mortality in acute myocardial infarction. Eur J Clin Invest.

[32] Sambola A, Ruiz-Meana M, Barba I (2017). Glycative and oxidative stress are associated with altered thrombus composition in diabetic patients with ST-elevation myocardial infarction. Int J Cardiol.

[33] Li JW, Chen YD, Chen WR, You Q, Li B, Zhou H, Zhang Y, Han TW (2017). Prognostic value of plasma DPP4 activity in ST-elevation myocardial infarction. Cardiovasc Diabetol.

[34] Wiemer M, Stoikovic S, Samol A, NOBORI 2 investigators (2017). Third generation drug eluting stent (DES) with biodegradable polymer in diabetic patients: 5 years follow-up. Cardiovasc Diabetol.

[35] Harada Y, Colleran R, Kufner S (2016). Five-year clinical outcomes in patients with diabetes mellitus treated with polymer-free sirolimus- and probucol-eluting stents versus second-generation zotarolimus-eluting stents: a subgroup analysis of a randomized controlled trial. Cardiovasc Diabetol.

