# Correction to: Thrombus aspiration in hyperglycemic ST-elevation myocardial infarction (STEMI) patients: clinical outcomes at 1-year follow-up

**DOI:** 10.1186/s12933-018-0804-y

**Published:** 2018-12-27

**Authors:** Celestino Sardu, Michelangela Barbieri, Maria Luisa Balestrieri, Mario Siniscalchi, Pasquale Paolisso, Paolo Calabrò, Fabio Minicucci, Giuseppe Signoriello, Michele Portoghese, Pasquale Mone, Davide D’Andrea, Felice Gragnano, Alessandro Bellis, Ciro Mauro, Giuseppe Paolisso, Maria Rosaria Rizzo, Raffaele Marfella

**Affiliations:** 1 Department of Medical, Surgical, Neurological, Aging and Metabolic Sciences, University of Campania “Luigi Vanvitelli”, Piazza Miraglia, 2, 80138 Naples, Italy; 2 Department of Biochemistry, Biophysics and General Pathology, University of Campania “Luigi Vanvitelli”, Naples, Italy; 3 Department of Cardiology, Hospital Cardarelli, Naples, Italy; 4 Department of Cardio-Thoracic and Respiratory Sciences, University of Campania “Luigi Vanvitelli”, Caserta, Italy; 5 Department of Mental Health and Public Medicine, Section of Statistic, University of Campania “Luigi Vanvitelli”, Naples, Italy; 6 Department of Cardiac Surgery, Hospital “SS Annunziata”, Sassari, Italy

## Correction to: Cardiovascular Diabetology (2018) 17:152 10.1186/s12933-018-0795-8

Following publication of the original article [1], the authors reported an error in Acknowledgment section. The last sentence should read as “All authors have read and approval the submission to Cardiovascular Diabetology”.

The authors wish to apologise for this error.
