# Supplementary figures and images for: Thrombus aspiration in hyperglycemic ST-elevation myocardial infarction (STEMI) patients: clinical outcomes at 1-year follow-up
Source: Cardiovasc Diabetol. 2018 Nov 29;17:152. doi: 10.1186/s12933-018-0795-8 (PMC6262961; doi:10.1186/s12933-018-0795-8)

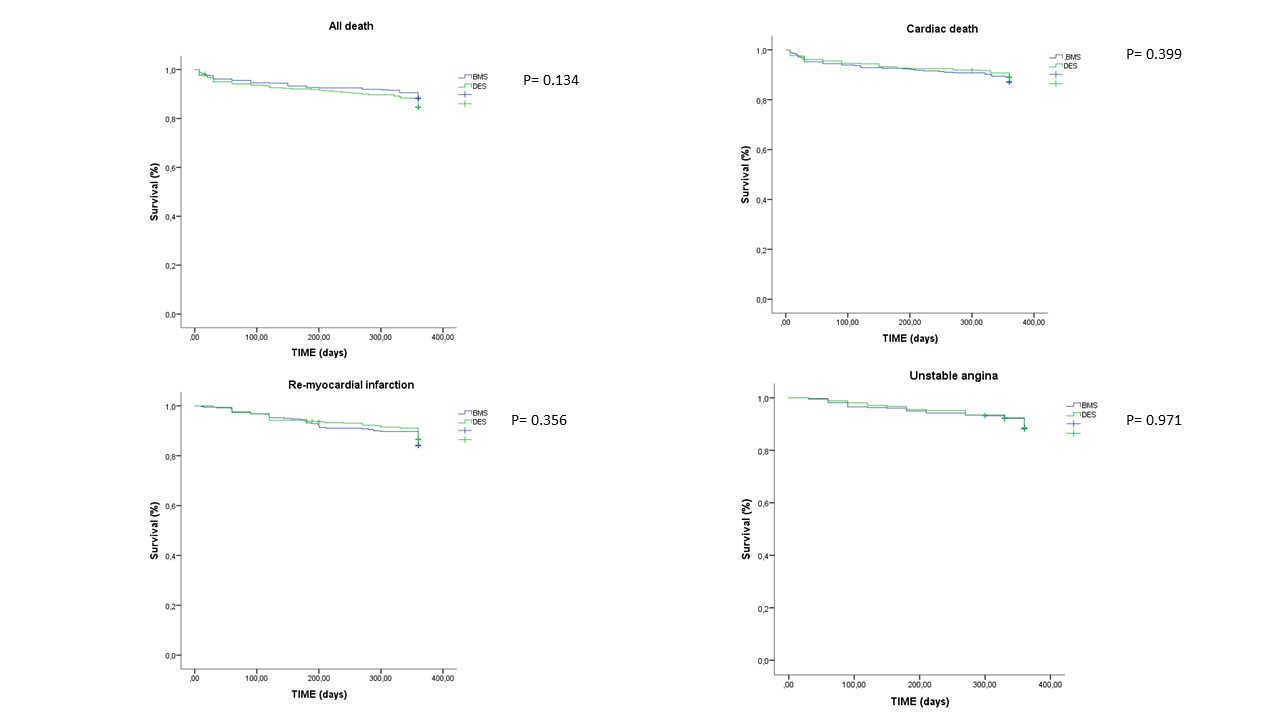

Supplement: Supplementary file 1 — Additional file 1. Kaplan Mayer curves in BMS and DES treated patients. [file 12933_2018_795_MOESM1_ESM.tif]
